# Habits and Psychological Factors Associated With Changes in Physical Activity Due to COVID-19 Confinement

**DOI:** 10.3389/fpsyg.2021.620745

**Published:** 2021-02-17

**Authors:** Eva León-Zarceño, Antonio Moreno-Tenas, Salvador Boix Vilella, Alejo García-Naveira, Miguel Angel Serrano-Rosa

**Affiliations:** ^1^Department of Behavioral Sciences and Health, Miguel Hernández University of Elche, Elche, Spain; ^2^Faculty of Humanities and Social Sciences, Isabel I University, Burgos, Spain; ^3^Colegio Oficial de la Psicología of Madrid, Madrid, Spain; ^4^Department of Psychobiology, Faculty of Psychology Valencia University, Valencia, Spain

**Keywords:** habits, COVID-19, coping, physical activity, psychological well-being

## Abstract

The confinement that COVID-19 has brought about has had a negative influence on people’s psychological health. However, this impact is not widespread throughout the population, and men and women may be affected differently and it is not known what protective factors may exist. In this sense, physical activity has classically been shown to be a habit associated with psychological health. The study aimed to analyze the impact of confinement on psychological health (psychological well-being, coping, emotions, and perception of daily difficulties), taking into account gender, and perceived changes in physical activity. After the project was approved by the University’s Ethics Commission, the participants, after signing the informed consent, completed the online questionnaires during the days from 6 to 20 April, the time when, in Spain, confinement was in place and the highest peak of deaths and infections from COVID-19 occurred. A total of 457 Spanish participants (247 men and 210 women) were evaluated in psychological well-being, in its adaptation to Spanish, in coping, with the Spanish adaptation of the COPE Inventory, in daily habits and difficulties (*ad hoc* questionnaire) and the level of physical activity they had (sedentary, active, and federated players) was recorded. Besides, the perceived change in physical activity due to confinement was recorded. The results showed that perceived emotions, difficulties for certain routines, psychological well-being, and coping differed according to sex. On the other hand, depending on the change in physical activity habits, it was observed that participants who increased their physical activity responded differently in the perception of emotions, and difficulties for routines and in psychological well-being. Finally, differences were also observed in most variables between sedentary, active, and federated participants. Results are discussed highlighting the importance of physical activity as a moderating factor of the impact of confinement.

## Introduction

COVID-19 disease has caused an alarming number of deaths and is a threat to society in terms of health, economy and healthy lifestyles (Ferreira-Júnior et al., 2020; Jaenes et al., 2020; Woods et al., 2020). The World Health Organization (WHO) declared a global pandemic by COVID-19 on March 11, 2020, and the most affected countries have limited freedom of movement within cities, generated restrictions, closed spaces (sports facilities, parks, and playgrounds, etc.), social distancing, hygiene measures, imposed a quarantine and established confinement of citizens to their homes (Lesser and Nienhuis, 2020; López-Bueno et al., 2020b; Mon-López et al., 2020).

Different studies during COVID-19 confinement, in countries such as China, Spain, Italy, Iran, United States, Turkey, Nepal, and Denmark, highlight that around 30% of the general population have suffered from anxiety, depression, psychological distress, adjustment disorder and insomnia, so the attention of researchers has focused on analyzing the factors that intensify or reduce negative emotional experience (Clemente-Suárez et al., 2020; Fu et al., 2020; Maugeri et al., 2020; Pieh et al., 2020; Rossi et al., 2020; Xionga et al., 2020).

Regarding gender differences studies tend to indicate that, during the confinement, women had a worse emotional response than men, with women presenting risk factors for poorer mental health in general (Pieh et al., 2020; Rossi et al., 2020), as well as in emotional states of irritability, anxiety and distress (Alsalhe et al., 2020; Fu et al., 2020; Jaenes et al., 2020; Mon-López et al., 2020; Xionga et al., 2020).

On one side, physical activity and sports, practiced regularly and in moderation, has been proved as a habit associated with people’s health and psychological well-being (García-Naveira and Locatelli, 2014), this being a key strategy for improving physical and mental health while the difficult situation of confinement by COVID-19 (Callow et al., 2020; Choi and Bum, 2020; Maugeri et al., 2020; Woods et al., 2020). Physical activity has been shown to be a good moderator of negative and unpleasant emotions (anger, fatigue, anxiety, and depression) (Alsalhe et al., 2020; Ingram et al., 2020; Jaenes et al., 2020), and has been associated with psychological well-being during confinement (Brand et al., 2020; Lesser and Nienhuis, 2020).

In Spain, confinement by the COVID-19 was decreed during the months of March to May 2020, and has led to a paralysis of sport and physical activity in the country, closing sports facilities, suspending training, competitions, major leagues and championships (Moscoso-Sánchez, 2020). Restrictive measures have limited the individual’s ability to engage in physical activity outdoors or in gyms, as well as regular training and competition in clubs, increasing the risks of chronic diseases related to a sedentary lifestyle and greater psychological vulnerability due to confinement (Ferreira-Júnior et al., 2020). Given their relevance, it is crucial to assess behavioral changes during periods of confinement, as active people suffer from the negative effects of the social context and those of the cessation of physical activity and sport (Brooks et al., 2020; Jaenes et al., 2020; Jukic et al., 2020).

During this period of isolation or quarantine, lifestyle and healthy habits have been modified due to individual and environmental differences (Brooks et al., 2020; Constant et al., 2020; Ingram et al., 2020; Jungmann and Witthöft, 2020; Liu et al., 2021), as is the case with the variation in frequency and duration of physical activity (Brand et al., 2020; Choi and Bum, 2020; Clemente-Suárez et al., 2020). Some studies warn that people in general reduced the length (days and hours) and intensity of physical activity during their confinement (Filho et al., 2020; Jimeìnez-Pavoìn et al., 2020; Mon-López et al., 2020; Xiang et al., 2020), mainly during the first week (changes in routines), although it may increase afterward (adaptation of routines) (López-Bueno et al., 2020b). COVID-19 home confinement has shown negative changes in population habits, both in terms of intensity levels of physical activity and eating patterns, being the last ones, unhealthier during confinement. Therefore, although isolation is a necessary measure to protect public health, physical activity and eating behaviors have been altered in a direction that undermines health (Ammar et al., 2020a). Recent research by COVID-19 suggests the existence of significant sleep problems and psychological disorders (e.g., stress, anxiety, and depression) associated with reduced movement and activity, as well as reduced social interaction; suggesting physical exercise at home, exergaming, dance with music and yoga, among the possible tactics to overcome the negative effects of confinement by recommending adults to do at least 150 min of moderate-intensity activity, and 75 min of vigorous activity per week divided into 5-7 sessions, and, reducing the volume of training in children and teenagers (Chtourou et al., 2020). A recent study determined that the levels of physical activity of Spanish population decreased significantly, up to 20%, in the first week of confinement for COVID-19, this reduction being especially relevant in men with a low educational level (López-Sánchez et al., 2020).

However, other studies analyze the pre-existing profile of people, classifying them as active or inactive in relation to physical activity. 40.5% of inactive subjects became even less active, 33% became more active and 26.5% remained as such, while 22.4% of active individuals became less active, 40.3% became more active and 37.3% remained as such (Lesser and Nienhuis, 2020).

Other studies indicate that, in general, less active people increased their physical activity (Brand et al., 2020; Schnitzer et al., 2020) and even those who exercised frequently before confinement tended to maintain or increase it (Brand et al., 2020).

In terms of changes in physical activity and emotional status during confinement, people who have remained physically active during confinement have better scores on quality of life, physical condition, psychological health, social relationships and environmental conditions than those who were inactive (Slimani et al., 2020). Those who were inactive before the pandemic, and slightly increased their frequency of exercise during the pandemic, reported no change in mood compared to those who remained inactive during the pandemic (Brand et al., 2020). These authors further noted that those who reduced their exercise frequency during the pandemic reported worse mood compared to those who maintained or increased their pre-pandemic exercise frequency.

Another relevant issue is the study of coping strategies used, such as adaptive emotional regulation which has been a buffer for reducing anxiety during a pandemic (Jungmann and Witthöft, 2020; Ye et al., 2020). Asmundson et al. (2020) conclude that there were no significant differences in the perceived effectiveness of coping strategies between the anxiety and mood disorder group vs. the non-pathological group during confinement, while Fu et al. (2020) note that passive coping with COVID-19 stress was relatively higher than before the pandemic. In addition, lack of fear control and cognitive avoidance are associated with poorer healthy lifestyle (Constant et al., 2020) with emotional intelligence and mood variables being predictors of training and athlete performance (Mon-López et al., 2020).

Finally, special mention should be made of the differentiation between high-performance athletes, other levels of competition and amateur practitioners in relation to confinement. One of the populations that could suffer most from confinement is sportsmen and women, especially professional ones, since their daily routines in which primary outdoor activities counteract the current situation of home confinement (Clemente-Suárez et al., 2020). For 2 months, high-performance sportsmen and women have been training at home, suffering great anxiety about their future, because the opportunity cost of a sports career is high (Moscoso-Sánchez, 2020). To keep training, without the certainty of a competition calendar is not an easy task. Constancy in training seems to have been an effective moderator of uncertainty, anxiety, stress, and in general of the negative emotions that had been so much anticipated (Jaenes et al., 2020), recommending emotional expression with other athletes as a strategy (Jaenes et al., 2020; Schinke et al., 2020), knowing that there may be some resistance from them not to be seen as weak in a culture that perceives athletes as mentally tough (Sherwin, 2017). Finally, studies indicate that male professional sportsmen trained more days and hours than semi-professional and amateur players (Mon-López et al., 2020).

Based on this review, there is a need for further research on the behavioral effects of the COVID-19 crisis during confinement. Specifically, this study focuses on gender differences (men and women), physical activity habits (no change, increase or decrease) and physical activity engagement (sedentary, physically active, and federated) in relation to the psychological impact (emotions, perception of difficulties, coping skills, and psychological well-being) that it has on people. We hypothesize that women will have poorer psychological health and that participants with a higher degree of physical activity will have higher scores on psychological health and coping strategies than those who are sedentary. Additionally, those participants who increased their physical activity habits during confinement will show higher scores on psychological health and coping.

## Materials and Methods

A cross-sectional online survey was conducted to evaluate physical exercise habits and psychological variables (psychological well-being and coping) during the COVID-19 pandemic.

### Design and Procedure

The study was approved by the Ethics Committees of the Miguel Hernandez University. All participants signed the informed consent. The participants completed the online questionnaires from April 6th to April 20th, while confinement was still in effect in Spain, and the highest peak of deaths and infections from COVID-19 occurred. The link to the survey was distributed on social networks (Telegram, WhatsApp, and email) to general population and athletes, all of legal age, for them to sign an informed consent, and to fill it out, and send it to their family or friends to fill it out as well. Afterward, generating groups by changes in physical activity frequency during confinement. Participants were asked about the daily frequency of different physical activities, before and during the confinement (walking, functional training, yoga/Pilates, bodybuilding, exercise bike, running, specific sport). To generate three different groups a difference between these two scores per activity.

Generating groups by changes in physical activity frequency during confinement: once differences were calculated for each activity, all differences were added up to obtain a value for each subject that could be 0 (no change in frequency of physical activity due to confinement), greater than 1 (increase in physical activity during confinement) or less than 1 (decrease in frequency of physical activity during confinement). Once the scores were obtained, the subjects were classified into the three groups indicated: no change in frequency of physical activity, increase in physical activity, and decrease in physical activity during confinement.

#### Participants

A total of 457 Spanish participants (247 men and 210 women) with an average age of 31.1 (dt = 11.37) and with different levels of exercise practice (sedentary, active and federated) were evaluated through an on-line questionnaire regarding their exercise habits before and during the period of confinement by COVID-19.

#### Variables and Instruments

Socio-demographic and sport variables were measured with an “*ad hoc*” questionnaire with answers on a Likert scale. This questionnaire evaluates daily habits of sports, emotions, as well as perception of difficulties during confinement, and the level of physical activity (sedentary, active, and federated). Through the “*ad hoc*” questionnaire, we inquired about their concerns and perception of difficulties in practice, changes in their training frequency, their emotional state due to confinement and how the difficulties due to the situation affected them in certain health and sports behaviors (“keeping a routine” or “falling asleep”).

##### Psychological well-being

This instrument measures were evaluated in psychological well-being, (SPWB-Van Dierendonck, 2004) in its adaptation to Spanish (Díaz et al., 2006) was used to psychological well-being and the Spanish version of this questionnaire has adequate psychometric properties (α = 83/α = 68). The EBP comprises 39 items, each of which contains six dimensions (Self-acceptance, positive relationships, autonomy, environment mastery, purpose in life, personal growth). Cronbach’s alpha in our sample was 0.90.

##### Coping

Coping strategies have been evaluated with Coping Inventory (COPE, Carver et al., 1989) with the Spanish adaptation (Crespo and Cruzado, 1997) through 60 items containing 15 coping strategies. This questionnaire measures the coping styles used by people when confronting a stressful event. The subscales that make up the questionnaire are Positive reinterpretation, Active coping, planning, Emotional Social Support, Instrumental Social support, Suppression competing activities, Religious, Acceptance, Restrain, Venting emotional, Behavioral disengagement, Denial, Mental disengagement, and Substance abuse. The Spanish version of the Coping Inventory to have adequate psychometric properties (α > 0.60). In our sample we obtained a α = 0.89.

#### Data Analysis

First, outliers were eliminated (+2.5 standard deviations) and the normality of the variables was calculated. No variable was normal. The variables were transformed into log10 and normality was recalculated. Since the data showed that the variables were not normal, the statistical analyses were carried out using non-parametric tests. The Mann-Whitney *U* parameter was used to compare the scores between the groups. The effect size is presented with the *r* parameter. All statistical analyses were performed with the SPSS 23 statistical package, with a significance level of 0.05.

## Results

### Sex Differences

Scores obtained during confinement differed between men and women. The characteristics of the study population are indicated in Table 1. Specifically, women had more negative thoughts about their physical condition [*U* = 14.265 (*Z* = −3.087), *p* = 0.002, *r* = 0.18] and about their health status as a trend (*p* = 0.061), women scored more in sadness [*U* = 30.837 (*Z* = −4.229), *p* < 0.001, *r* = 0.19], tension [*U* = 29.618 (*Z* = −3.221), *p* = 0.001, *r* = 0.15] and support by others [*U* = 30.967 (*Z* = −3.769), *p* < 0.001, *r* = 0.17] and lower energy scores [*U* = 22.49 (*Z* = −2.521), *p* = 0.012, *r* = 0.11], compared to men. Also, women reported greater difficulty in training than men. Complementarily, they had greater difficulty following feeding [*U* = 28.491 (*Z* = −3.769), *p* = 0.05, *r* = 0.08], sleeping [*U* = 28.764 (*Z* = −3.769), *p* < 0.001, *r* = 0.09], and following daily routines [*U* = 28.761 (*Z* = −3.769), *p* = 0.038, *r* = 0.09]. Complementarily, men scored higher on the positive perception of confinement [*U* = 22.703 (*Z* = −3.769), *p* < 0.001, *r* = 0.11].

**TABLE 1:** Characteristics of the study population.

***N* = 457**	***n* (%)**	**Mean (SD)**
Age		31.10 (11.37)
**Gender**		
Men	247 (51)	
Women	210 (46)	
**Shared housing space**		
Alone	25 (5.5)	
With couple	99 (21.7)	
Family	325 (71.1)	
Others	8 (1.7)	
Number of people living together*		
0	21 (4.6)	
1	72 (15.8)	
2	71 (15.5)	
+3	147 (32.2)	
Type of Housing*		
Flat	224 (49)	
Duplex	30 (6.6)	
House with plot	56 (12.3)	
**Have a dog**		
Yes	324 (70.9)	
No	133 (29.1)	

*147 missing values.

In the questionnaires, women used significantly more emotional support seeking coping strategies [*U* = 14.674 (*Z* = −3.769), *p* < 0.001, *r* = 0.19], as well as venting [*U* = 16.290 (*Z* = −3.769), *p* < 0.001, *r* = 0.30] and mental disengagement [*U* = 13.898 (*Z* = −3.769), *p* < 0.001, *r* = 0.13], compared to men. On the other hand, men score more on psychological well-being, specifically on autonomy [*U* = 10.302 (*Z* = −3.769), *p* < 0.001, *r* = 0.12], environmental mastery [*U* = 10.411 (*Z* = −3.769), *p* = 0.04, *r* = 0.11], and purpose in life [*U* = 10.439 (*Z* = −3.769), *p* = 0.044, *r* = 0.11] (see Table 2).

**TABLE 2:** Mean and standard deviation (SD) of men and women in the measured variables after 1 month of confinement.

	**Men**		**Women**		**Significant**
	***N* = 247**		***N* = 210**		**differences**
	**Mean**	**SD**	**Mean**	**SD**	
**Mood**					
Confinement feelings	1.68	1.04	1.81	0.93	n.s.
Health thoughts	0.68	0.62	0.79	0.63	t
Physical condition thoughts	0.86	0.87	1.13	0.86	*
Sadness	0.83	0.80	1.15	0.83	*
Tension	0.89	0.82	1.18	0.93	*
Irritability	1.20	0.99	1.12	0.90	n.s.
Energy	1.97	1.16	1.68	1.14	*
Fatigue	0.87	0.93	0.98	0.95	n.s.
Others support	1.67	1.23	2.09	1.11	*
Positive confinement perception	1.21	0.40	1.12	0.33	*
**Perceived difficulties**					
Initial Training Difficulty	4.10	3.18	4.74	3.27	n.s.
Current training difficulty	3.89	2.82	4.54	2.99	n.s.
Sleep difficulties	1.36	1.33	1.61	1.36	*
Feeding difficulties	1.11	1.22	1.27	1.14	*
Daily Routines difficulties	1.27	1.26	1.51	1.31	*
Training plan difficulties	1.38	1.28	1.44	1.31	n.s.
Reaching aims difficulties	1.51	1.33	1.64	1.36	n.s.
**Coping**					
COPE_Positive_ reinterpretation	7.73	2.57	7.79	2.40	n.s.
COPE_Active coping	6.39	2.15	6.25	2.23	n.s.
COPE_planning	6.21	2.77	6.09	2.36	n.s.
COPE_Emotional Social Support	5.74	3.03	6.84	2.87	*
COPE_Instrumental Social support	5.25	2.51	5.70	2.52	n.s.
COPE_Suppression competing activities	5.05	2.18	4.88	1.99	n.s.
COPE_Religious	1.15	2.17	1.38	2.39	n.s.
COPE_Acceptance	6.65	2.39	6.88	2.58	n.s.
COPE_Restrain	3.95	2.06	4.63	2.41	n.s.
COPE_Venting emotional	3.87	2.23	5.19	2.36	*
COPE_Behavioral disengagement	1.77	1.93	1.83	2.01	n.s.
COPE_Denial	1.47	1.81	1.41	1.79	n.s.
COPE_Mental disengagement	5.00	2.06	4.86	2.07	*
COPE_Substance abuse	0.56	1.44	0.57	1.83	n.s.
COPE_Humor	4.81	3.60	4.32	2.92	n.s.
**Psychological well being**					
PWB_Self_acceptance	23.01	3.85	22.52	3.60	n.s.
PWB_Positive_relations	24.39	3.64	24.07	4.36	n.s.
PWB_Autonomy	29.97	4.42	28.75	4.63	*
PWB_Environmental_ mastery	23.44	3.11	22.59	3.14	*
PWB_Personal_growth	27.76	3.69	27.99	3.01	n.s.
PWB_Life_purpose	24.04	3.95	23.21	3.87	*
**Habits changes**					
Physical_Activity_ Frequency_change (PAFch)	−0.008	4.05	0.171	5.53	n.s.
Physical_Activity_ Duration_change (PADch)	−2.44	3.41	−1.70	3.10	n.s.

n.s., non-significant; *, significant; t, trend.

### Physical Activity Habit Changes

When physical activity frequency habit change is analyzed there were differences between participants that increased or decreased or did not change their habits (see Table 3).

**TABLE 3:** Mean and standard deviation (SD) of participants depending on the increase, decrease or no change in their daily frequency of physical activity in the measured variables after 1 month of confinement.

	**No change**		**Frequency increase**		**Frequency decrease**		**Significant**
	**(*n* = 63)**		**(*n* = 216)**		**(*n* = 178)**		**differences**
	**Mean**	**SD**	**Mean**	**SD**	**Mean**	**SD**	
**Mood**							
Confinement feelings	1.62	0.92	1.83	0.99	1.67	1.02	1 vs. 2
Health thoughts	0.72	0.63	0.73	0.62	0.73	0.64	n.s.
Physical condition thoughts	0.86	0.67	1.03	0.87	1.04	0.94	n.s.
Sadness	0.87	0.82	1.01	0.81	0.97	0.85	n.s.
Tension	0.98	0.79	1.11	0.97	0.93	0.81	n.s.
Irritability	1.00	0.81	1.11	0.95	1.27	0.98	n.s.
Energy	1.71	1.12	1.72	1.19	2.02	1.12	1 vs. 2 0 vs. 2
Fatigue	0.77	0.84	0.99	0.97	0.89	0.94	n.s.
Others support	1.76	1.16	1.85	1.23	1.92	1.18	n.s.
Positive confinement perception	1.17	0.37	1.20	0.40	1.13	0.33	1 vs. 2
**Perceived difficulties**							
Initial training difficulty	3.92	3.07	4.74	3.34	4.15	3.13	n.s.
Current training difficulty	4.05	2.81	4.81	3.05	3.49	2.61	1 vs. 2
Sleep difficulties	1.40	1.30	1.59	1.37	1.35	1.34	n.s.
Feeding difficulties	0.95	1.06	1.38	1.23	1.03	1.14	1 vs. 2 0 vs. 1
Daily routines difficulties	1.33	1.25	1.53	1.33	1.21	1.23	1 vs. 2
Training plan difficulties	1.16	1.24	1.73	1.36	1.11	1.12	0 vs. 1 1 vs. 2
Reaching aims difficulties	1.29	1.32	1.77	1.40	1.42	1.26	1 vs. 2 0 vs. 1
**Coping**							
COPE_Positive_reinterpretation	7.09	2.65	7.46	2.36	8.42	2.44	0 vs. 2
COPE_Active coping	6.02	2.28	6.10	1.99	6.71	2.37	n.s.
COPE_planning	5.63	2.32	6.00	2.53	6.54	2.64	n.s.
COPE_Emotional Social Support	6.09	3.24	6.16	2.87	6.66	3.06	n.s.
COPE_Instrumental Social support	5.19	2.57	5.45	2.39	5.66	2.69	n.s.
COPE_Suppression competing activities	5.14	2.25	4.66	1.89	5.30	2.22	1 vs. 2
COPE_Religious	1.42	2.60	1.27	2.29	1.23	2.17	n.s.
COPE_Acceptance	6.33	2.72	6.48	2.36	7.34	2.50	1 vs. 2
COPE_Restrain	3.49	2.14	4.53	2.28	4.34	2.27	n.s.
COPE_Venting emotional	4.67	2.31	4.52	2.39	4.62	2.45	n.s.
COPE_Behavioral disengagement	2.00	1.97	1.88	2.09	1.62	1.79	n.s.
COPE_Denial	1.28	1.57	1.65	1.96	1.21	1.61	n.s.
COPE_Mental disengagement	4.77	1.90	5.00	2.21	4.89	1.94	0 vs. 1
COPE_Substance abuse	0.65	1.66	0.66	1.97	0.42	1.11	n.s.
COPE_Humor	4.37	3.68	4.62	3.22	4.51	3.16	n.s.
**Psychological well being**							
PWB_Self_acceptance	23.16	2.60	22.57	3.96	22.83	3.76	n.s.
PWB_Positive_relations	25.09	2.91	23.91	4.08	24.30	4.32	n.s.
PWB_Autonomy	29.19	4.04	29.34	4.67	29.33	4.65	n.s.
PWB_Environmental_mastery	22.91	2.77	22.84	3.15	23.20	3.29	n.s.
PWB_Personal_growth	27.53	3.35	27.77	3.37	28.18	3.30	n.s.
PWB_Life_purpose	23.88	3.31	23.31	4.02	23.88	3.99	n.s.

*0, no daily training activity changes; 1, increasing daily training activities; 2, decreasing daily training activities; n.s., no significant differences.*

#### Differences Within Participants With no Change in Frequency and the Increased/Decreased Frequency

The group that increased frequency scored higher in following feeding [*U* = 5.505 (*Z* = −2.393, *p* = 0.017, *r* = 0.14] and training routines [*U* = 5.154 (*Z* = −3.006), *p* = 0.003, *r* = 0.17] and having goals [*U* = 5.426 (*Z* = −2.505), *p* = 0.012, *r* = 0.14] than the group that did not change their weekly workout frequency. Likewise, this group that did not modify its frequency had different scores in mental disengagement than the group that increases its frequency [*U* = 2.551 (*Z* = −2.445), *p* = 0.014, *r* = 0.17].

In addition, the group that reduced their physical activity differed significantly in perceived energy [*U* = 4.616 (*Z* = −2.152), *p* = 0.031, *r* = 0.13] positive coping in comparison to the subjects in the group that does not change their training frequency [*U* = 1.776 (*Z* = −2.678), *p* = 0.007, *r* = 0.21].

#### Differences Between Increased Frequency and Decreased Frequency

A trend toward significance has been obtained among participants who increased their physical activity frequency that are more affected by the confinement situation than those who reduced their activity frequency (*p* = 0.06). On the other hand, it was observed that participants who reduced their frequency have higher scores in perceived energy compared to groups who increase their activity [*U* = 22.135 (*Z* = −2.664), *p* = 0.008, *r* = 0.13].

In addition, those who increased their frequency have greater difficulty training than participants who reduced their activity frequency [*U* = 14.661 (*Z* = −4.082), *p* < 0.001, *r* = 0.20]. Also, the group that increases their frequency has greater difficulty following feeding patterns [*U* = 16.130 (*Z* = −2.868), *p* = 0.004, *r* = 0.14], the daily routines [*U* = 16.652 (*Z* = −2.363), *p* = 0.018, *r* = 0.11], training plan [*U* = 14.282 (*Z* = −4.545), *p* < 0.001, *r* = 0.22], or having daily goals [*U* = 16.565 (*Z* = −2.424), *p* = 0.015, *r* = 0.12] compared to those who reduced their frequency. However, this group scored significantly higher on seeing confinement as something positive, than the group that reduces its activity frequency [*U* = 17.035 (*Z* = −1.993), *p* = 0.046, *r* = 0.10].

Finally, when coping styles were studied, it was observed that those who increased their activity frequency had lower scores on positive coping [*U* = 10.636 (*Z* = −2.988), *p* = 0.003, *r* = 0.18], suppression of competitive activities [*U* = 10.142 (*Z* = −2.630), *p* = 0.009, *r* = 0.13], and acceptance [*U* = 10.547 (*Z* = −2.841), *p* = 0.005, *r* = 0.17] compared to the group that reduced their physical activity.

### Comparison Between Sedentary, Physically Active, and Federated Players Participants

When the participants’ responses were analyzed according to their degree of involvement with physical activity, the following results were found (see Table 4).

**TABLE 4:** Mean and standard deviation (SD) of participants depending on the physical activity before confinement (Sedentary, Physically active and Federated players) in the measured variables after 1 month of confinement.

	**Sedentary**		**Physically active**		**Federated Players**		**Significant differences**
	**Mean**	**SD**	**Mean**	**SD**	**Mean**	**SD**	
**Mood**							
Confinement feelings	1.51	1.05	1.72	0.88	1.83	1.05	1 vs. 3
Health thoughts	0.71	0.62	0.73	0.65	0.74	0.62	n.s.
Physical condition thoughts	0.90	0.85	1.04	0.83	1.03	0.98	n.s.
Sadness	0.94	0.81	1.06	0.82	0.93	0.85	n.s.
Tension	1.01	0.90	1.19	0.86	0.91	0.89	2 vs. 3
Irritability	0.87	0.86	1.01	0.80	1.36	1.04	1 vs. 3 2 vs. 3
Energy	1.72	1.11	1.57	1.07	2.06	1.20	1 vs. 3 2 vs. 3
Fatigue	0.99	0.92	0.86	0.94	0.94	0.96	n.s.
Others support	2.06	1.13	2.06	1.19	1.66	1.20	1 vs. 3 2 vs. 3
Positive confinement perception	1.15	0.36	1.16	0.37	1.18	0.38	n.s.
**Perceived difficulties**							
Initial training difficulty	5.94	3.14	4.23	3.09	4.03	3.24	1 vs. 2
Current training difficulty	4.92	3.12	4.11	3.02	4.02	2.75	1 vs. 3
Sleep difficulties	1.49	1.30	1.41	1.35	1.51	1.38	n.s.
Feeding difficulties	1.08	1.08	1.16	1.18	1.23	1.23	n.s.
Daily routines difficulties	1.49	1.26	1.49	1.33	1.27	1.27	n.s.
Training plan difficulties	1.20	1.26	1.53	1.37	1.39	1.25	n.s.
Reaching aims difficulties	1.25	1.28	1.62	1.35	1.63	1.36	1 vs. 2 1 vs. 3
**Coping**							
COPE_Positive_reinterpretation	8.13	2.48	7.58	2.53	7.81	2.38	n.s.
COPE_Active coping	6.55	2.27	6.12	2.21	6.49	2.09	n.s.
COPE_planning	6.27	2.54	5.79	2.56	6.74	2.49	2 vs. 3
COPE_Emotional Social Support	6.61	2.69	6.22	3.19	6.31	2.88	n.s.
COPE_Instrumental Social support	5.70	2.24	5.24	2.66	5.80	2.48	n.s.
COPE_Suppression competing activities	5.39	2.02	4.80	2.12	4.89	2.06	n.s.
COPE_Religious	1.34	2.20	1.23	2.25	1.33	2.47	n.s.
COPE_Acceptance	7.00	2.52	6.94	2.45	6.24	2.53	2 vs. 3
COPE_Restrain	4.51	2.14	4.33	2.36	4.13	2.24	n.s.
COPE_Venting emotional	5.08	2.23	4.62	2.51	4.05	2.21	1 vs. 3
COPE_Behavioral disengagement	1.79	1.83	1.98	2.06	1.46	1.90	2 vs. 3
COPE_Denial	1.62	1.82	1.45	1.81	1.25	1.75	n.s.
COPE_Mental disengagement	4.90	2.04	4.98	2.10	4.85	2.05	n.s.
COPE_Substance abuse	0.41	1.12	0.69	1.95	0.48	1.41	n.s.
COPE_Humor	4.80	3.30	4.28	3.17	4.85	3.38	n.s.
**Psychological well being**							
PWB_Self_acceptance	22.46	3.79	22.40	3.69	23.70	3.63	1 vs. 3 2 vs. 3
PWB_Positive_relations	23.68	4.49	24.34	3.89	24.44	3.96	n.s.
PWB_Autonomy	29.03	4.68	29.21	4.66	29.80	4.29	n.s.
PWB_Environmental_mastery	22.61	2.78	22.84	3.25	23.59	3.22	1 vs. 3 2 vs. 3
PWB_Personal_growth	28.34	3.15	27.65	3.46	27.95	3.27	
PWB_Life_purpose	23.72	3.46	23.00	4.06	24.68	3.84	1 vs. 3 2 vs. 3
**Habits change**							
Physical_Activity_Frequency_change (PAFch)	2.55	4.99	−0.93	4.69	0.01	4.53	1 vs. 2 1 vs. 3
Physical_Activity_Duration_change (PADch)	−0.25	3.38	−2.73	3.10	−2.23	3.20	1 vs. 2 1 vs. 3

*1, sedentary; 2, physically active; 3, federated players; n.s, no significant differences.*

#### Sedentary vs. Physically Active Participants (1 vs. 2)

Sedentary participants significantly increased their physical activity frequency [*U* = 3.607 (*Z* = −4.442), *p* < 0.001, *r* = 0.29] and duration [*U* = 6.275 (*Z* = −6.403), *p* < 0.001, *r* = 0.08] compared to physically active participants. In addition, sedentary participants had less difficulty in having daily objectives during confinement [*U* = 6.567 (*Z* = −1.949), *p* = 0.05, *r* = 0.12], although at the beginning of the confinement, they had more difficulty training than physically active participants [*U* = 3.988 (*Z* = −3.636), *p* < 0.001, *r* = 0.23].

#### Sedentary vs. Federated Players Participants (1 vs. 3)

When sedentary were compared with federated players participants, the former significantly increased the frequency [*U* = 5.606 (*Z* = −3.845), *p* < 0.001, *r* = 0.22] and duration [*U* = 5.762 (*Z* = −5.046), *p* < 0.001, *r* = 0.20] of physical activity compared with the latter, which remained stable in terms of frequency and moderately reduced the duration of training.

On the other hand, the confinement situation affected negatively and significantly more the federated players compared to the sedentary ones [*U* = 9.374 (*Z* = −2.231), *p* = 0.026, *r* = 0.12]. On the other hand, sedentary participants presented lower irritation scores [*U* = 9.36 (*Z* = −3.405), *p* < 0.001, *r* = 0.20] and felt more supported by others [*U* = 6.430 (*Z* = −2.561), *p* = 0.010, *r* = 0.14] than federated. In contrast, federated players had significantly higher energy scores [*U* = 9.298 (*Z* = −2.078), *p* = 0.038, *r* = 0.12] and had less difficult to train than sedentary individuals [*U* = 5.390 (*Z* = −2.139), *p* = 0.032, *r* = 0.24]. Regarding the difficulties experienced by the participants, it was found that the sedentary subjects had less difficulty in having objectives than the federated ones [*U* = 9.30 (*Z* = −2.080), *p* = 0.038, *r* = 0.12].

Finally, with respect to the differences obtained when comparing the coping and psychological well-being scores, it was obtained that sedentary participants had significantly higher scores on the emotional venting scale [*U* = 2.116 (*Z* = −2.749), *p* = 0.006, *r* = 0.22]. However, federated scored significantly higher on self-acceptance [*U* = 3.383 (*Z* = −2.032), *p* = 0.042, *r* = 0.16], environmental mastery [*U* = 3.460 (*Z* = −2.325), *p* = 0.020, *r* = 0.18] and purpose in life [*U* = 3.350 (*Z* = −1.911), *p* = 0.056, *r* = 0.15] compared to sedentary participants.

#### Physical Active vs. Federated Players Participants (2 vs. 3)

It has been obtained that there were no significant differences in the change of total frequency nor in the total duration of training, maintaining both groups a similar level of frequency of physical activity with respect to the previous confinement period and slightly reducing the duration of training.

However, the physically active group scored significantly higher on perceived tension [*U* = 14.410 (*Z* = −3.441), *p* = 0.001, *r* = 0.17] and irritability [*U* = 20.180 (*Z* = −3.105), *p* = 0.0.002, *r* = 0.16] than the federated group. In addition, the federated players group scored higher on perceived energy [*U* = 22.210 (*Z* = −3.936), *p* < 0.001, *r* = 0.20]. On the other hand, the active group felt more supported by the others than the federated players group [*U* = 14.415 (*Z* = −3.429), *p* = 0.001, *r* = 0.17].

When coping and well-being questionnaires were analyzed, results showed that the federated subjects significantly use the planning coping strategy [*U* = 7.675 (*Z* = −2.533), *p* = 0.011, *r* = 0.16], but have less acceptance [*U* = 5.378 (*Z* = −2.029), *p* = 0.042, *r* = 0.13] and less behavioral disengagement [*U* = 5.386 (*Z* = −2.040), *p* = 0.040, *r* = 0.13] than the physically active subjects. Finally, in the psychological well-being questionnaire, federated participants scored more on self-acceptance [*U* = 7.812 (*Z* = −2.796), *p* = 0.005, *r* = 0.18] and environmental mastery [*U* = 7.410 (*Z* = −2.002), *p* = 0.045, *r* = 0.12] and less on purpose in life [*U* = 7.969 (*Z* = −3.108), *p* = 0.002, *r* = 0.20], compared to the physically active group.

## Discussion

During the health alert by COVID-19 many sectors of society have been affected and have had to adapt their lives and habits to the new context. The first objective of this work was to analyze whether there were gender differences in the population confined to emotions, perception of training difficulties, coping strategies and psychological well-being.

In relation to this objective, the results of the present study confirm the existence of statistically significant differences between men and women, in which women present higher scores than men in negative thoughts (state of health, physical condition, and about confinement), negative emotions (sadness, tension, and energy) and worse psychological well-being (less autonomy, environmental dominance and purpose in life). These data are in line with prior research indicating that women have a worse emotional response and mental health than men during confinement (Alsalhe et al., 2020; Fu et al., 2020; Jaenes et al., 2020; López-Bueno et al., 2020c; Mon-López et al., 2020; Pieh et al., 2020; Rossi et al., 2020; Xionga et al., 2020). Some studies have found that home confinement by COVID-19 had a negative effect on both psychological well-being and mood by increasing depressive symptoms and increasing psychosocial stress caused by home confinement. Interdisciplinary intervention is needed in addition to promoting an active and healthy confinement lifestyle to mitigate this high risk of mental disorders (Ammar et al., 2020c).

In addition, this study shows that women perceive greater difficulty in leading a healthy life in general in this situation (daily routines, eating and sleeping patterns), issues that may be related to the worst emotional response during confinement. In addition, women are more focused on coping strategies that focus on emotional support (letting go and mental disconnection), issues that may be limited by social restrictions and alienation.

Therefore, gender differences are an important area of study during periods of confinement, in this case derived from COVID-19, requiring special attention for the female population, developing coping strategies that are adaptive to the situation and to the perceived difficulties in relation to certain healthy habits, with the aim of improving their emotional state and psychological well-being. In addition to greater attention to women, it would be interesting if the different national and international bodies agreed to implement healthy recommendations for each subgroup, for example, older or sick women (Bentlage et al., 2020), taking advantage of the technological tools available in homes (Ammar et al., 2020b).

The second objective analyzed was the changes in the frequency of physical activity (increase, maintenance, or decrease) on emotions, perceived difficulties, coping and psychological well-being.

It is perceived that there is a variation in physical activity during confinement (444 people; 86.3%), as indicated in previous works (Brand et al., 2020; Choi and Bum, 2020; Clemente-Suárez et al., 2020). In the present study, there is mainly an increase in physical activity (266 participants; 47.26%), although there is also a high number of people who decrease their practice (178 individuals; 38.51%), an issue that is reinforced by studies that perceive an increase in physical activity (Brand et al., 2020; Schnitzer et al., 2020) and those who obtain a decrease in practice (Filho et al., 2020; Jimeìnez-Pavoìn et al., 2020; Lesser and Nienhuis, 2020; López-Bueno et al., 2020a; Mon-López et al., 2020; Xiang et al., 2020; Ammar et al., 2021), although a period of time is required to adapt to the new context and routines (López-Bueno et al., 2020b).

This change and variation in physical activity depends on certain pre-existing individual variables (lifestyle, healthy habits, personal variables, etc.) and contextual variables (sports equipment, space in the home, doing physical activity accompanied, with an online trainer, etc.), as indicated by other authors (Brooks et al., 2020; Constant et al., 2020; Ingram et al., 2020; Jungmann and Witthöft, 2020; Liu et al., 2021).

Secondly, the data obtained indicate that when participants increase their physical activity, they perceive greater difficulties in training, in following their eating patterns, training plan, and daily objectives. Furthermore, they are more affected by the confinement situation, although they perceive confinement as positive and use mental disconnection coping strategies.

This person profile may be concerned about the limitations (personal and contextual) of being locked in at home and perceive the need for and benefit of physical activity during confinement. These participants require greater order and organization to resolve the difficulties they perceive, although this does not prevent them from increasing their physical activity, which makes the confinement more manageable and helps them to disconnect from the worries. On the other hand, the group that reduced their physical activity was perceived as having more energy and positive coping (suppression of competitive activities and acceptance), which could represent accepting the situation and saving resources for day-to-day life, at least for a period of time, especially if they do not have the resources to be physically active or if they decide not to.

In their case, the confinement of sportsmen and women was more emotionally affecting than that of sedentary or even physically active sportsmen and women. Thus, the athletes showed high scores in irritation and felt less support than the sedentary ones, although they were, in turn, less tense and irritated than the physically active ones. The cancelation of all the competitions, together with the uncertainty of returning to them, meant that they experienced negative emotional changes as well as difficulties in setting goals. These findings are in line with other works that stress the important role played by emotions in competitive athletes (Jaenes et al., 2020; Jukic et al., 2020). To cope with this situation, athletes have used active coping strategies such as planning, and have accepted the situation despite having lost their purpose in life, a logical consequence of losing the possibility of competing and achieving their goals. Undoubtedly, the role that coping strategies have played has been relevant as a moderator between the stressful event of a pandemic and well-being (Rettie and Daniels, 2020; Ye et al., 2020).

Therefore, there are several future challenges in minimizing the psychological impact of confinement during a pandemic. Firstly, it will be necessary to design specific psychological interventions to improve coping strategies (Pfefferbaum and North, 2020), focusing, among others, on interventions aimed at improving cognitive flexibility, as recommended by other researchers, facilitating adjustment and adaptation to changes in athletes’ lives due to the pandemic (Clemente-Suárez et al., 2020). Another future challenge will focus on finding out to what extent the practice of physical activity is producing psychological benefits in times of confinement, and on what specific variables, given that the emotional effects of such activity and physical exercise habits appear to be moderated by numerous variables. Future lines of work include the evaluation of new psychological variables that could shed light on the data obtained, such as the type of motivation to practice among participants, the design of specific intervention programs according to the level of practice, as well as analyzing the psychological impact of the pandemic among elite athletes, taking into account gender differences. In view of the results obtained in this study, it would be advisable to monitor the emotional states and healthy habits of the athletes, both daily, weekly and even monthly, following the recommendations of other works, that have suggested that exercises should be adapted to the participant’s level of physical condition and a progressive model of training intensity and volume should be used, preferably monitored by telephone applications and portable sensors (Chtourou et al., 2020). It is also essential to generate spaces for communication so that the technical body and the team members themselves can share their state at an emotional, cognitive and behavioral level, generating feelings of identity and affiliation. Another recommendation is that, during times of confinement, professional attention should be received from the psychologist, both as a preventive measure, psychological attention and mental preparation, since it is essential that the athlete be prepared and psychologically balanced for when training and competition resume. Finally, given the cancelations and changes in competition calendars, it is important to help the athlete to plan and organize his/her life and sporting activity, despite the uncertainty of the situation, focusing on the “here and now” and regulating the negative emotions that may arise (e.g., relaxation, positive internal self-dialogue and self-instruction, engaging in enjoyable activities, social support, etc.) by helping them to set short-term goals (e.g., for each day or week). In short, if we are to make effective and healthy interventions in exercise habits in future confinement, it will be necessary to understand what difficulties they encounter in alleviating them. Similarly, we need to consider where the differences lie between people who, prior to the pandemic, did not engage in any form of physical activity and those who did. Without doubt, complementing this information with the inclusion of qualitative variables can broaden the possibilities of understanding the results obtained in the face of such an unusual event as a confinement.

Among the limitations of this study, we can include the absence of sociodemographic variables such as income or previous psychological diagnoses, such as anxiety or depression, which could affect the results obtained since they could be associated with the emotional impact of the pandemic (Fu et al., 2020; Jungmann and Witthöft, 2020; Pieh et al., 2020; Xionga et al., 2020). In addition, other possible limitation would be of the study was that the sample through convenience sampling, which could lead to a bias regarding the representativeness of the sample. Another limitation is the size of the effect obtained, which limits possible practical interpretations of the results. This research is cross-sectional, which makes it impossible to follow up on the participants once the confinement has ended.

The results found in this research about the changes produced in the exercise habits and their relationship with coping and emotions will allow the design of psychological interventions that take into account both gender differences and the different levels of involvement in physical activity given the different repercussions on the psychological variables analyzed.

## Data Availability Statement

The raw data supporting the conclusions of this article will be made available by the authors, without undue reservation.

## Ethics Statement

The study was reviewed and approved by the Ethics Committee of Miguel Hernandez University. The participants provided their written informed consent to participate in this study.

## Author Contributions

EL-Z and AM-T developed the design of the research. AG-N reviewed the literature. MS-R and SB performed the data analyses. All authors participated in the development of the study, contributed in writing the first draft and reviewed the final draft. The manuscript has been approved by all authors.

## Conflict of Interest

The authors declare that the research was conducted in the absence of any commercial or financial relationships that could be construed as a potential conflict of interest.

## References

[B1] Alsalhe T. Aljaloud S. Chalghaf N. Guelmami N. Alhazza D. Azaiez F. (2020). Moderation effect of physical activity on the relationship between fear of covid-19 and general distress: a pilot case study in Arabic countries. *Front. Psychol.* 11:2381. 10.3389/fpsyg.2020.570085 33071900 PMC7539623

[B2] Ammar A. Brach M. Trabelsi K. Chtourou H. Boukhris O. Masmoudi L. (2020a). Effects of COVID-19 home confinement on eating behavior and physical activity: results of the ECLB-COVID19 international online survey. *Nutrients* 12:1583. 10.3390/nu12061583 32481594 PMC7352706

[B3] Ammar A. Chtourou H. Boukhris O. Trabelsi K. Masmoudi L. Brach M. (2020b). COVID-19 home confinement negatively impacts social participation and life satisfaction: a worldwide multicenter study. *Int. J. Environ. Res. Public Health* 17:6237. 10.3390/ijerph17176237 PMC7503681 32867287

[B4] Ammar A. Mueller P. Trabelsi K. Chtourou H. Boukhris O. Masmoudi L. (2020c). Psychological consequences of COVID-19 home confinement: the ECLB-COVID19 multicenter study. *PLoS One* 15:e0240204. 10.1371/journal.pone.0240204 33152030 PMC7643949

[B5] Ammar A. Trabelsi K. Brach M. Chtourou H. Boukhris O. Masmoudi L. (2021). Effects of home confinement on mental health and lifestyle behaviors during the COVID-19 outbreak: insight from the ECLB-COVID19 multicenter study. *Biol. Sport* 38 9–21. 10.5114/biolsport.2020.96857 PMC7996377 33795912

[B6] Asmundson G. Paluszek M. Landry C. Rachor G. McKay D. Taylor S. (2020). Do pre-existing anxiety-related and mood disorders differentially impact COVID-19 stress responses and coping? *J. Anxiety Disord.* 74:102271. 10.1016/j.janxdis.2020.102271 32673930 PMC7342169

[B7] Bentlage E. Ammar A. How D. Ahmed M. Trabelsi K. Chtourou H. (2020). Practical recommendations for maintaining active lifestyle during the COVID-19 pandemic: A systematic literature review. *Int. J. Environ. Res. Public Health* 17:6265. 10.3390/ijerph17176265 32872154 PMC7503956

[B8] Brand R. Timme S. Nosrat S. (2020). When pandemic hits: exercise frequency and subjective well-being during COVID-19 pandemic. *Front. Psychol.* 11:2391. 10.3389/fpsyg.2020.570567 33071902 PMC7541696

[B9] Brooks S. K. Webster R. K. Smith L. E. Woodland L. Wessely S. Greenberg N. (2020). The psychological impact of quarantine and how to reduce it: rapid review of the evidence. *Lancet* 395 912–920. 10.1016/S0140-6736(20)30460-8 32112714 PMC7158942

[B10] Callow D. Arnold-Nedimala N. Jordan L. Pena G. Won J. Woodard J. (2020). The mental health benefits of physical activity in older adults survive the COVID-19 pandemic. *Am. J. Geriatric Psychiatry* 28 1046–1057. 10.1016/j.jagp.2020.06.024 PMC7831892 32713754

[B11] Carver C. S. Scheier M. F. Weintraub J. K. (1989). Assessing coping strategies: a theoretically based approach. *Journal of Person. Social Psychol.* 56 267–283. 10.1037/0022-3514.56.2.267 2926629

[B12] Choi C. Bum C. H. (2020). Changes in the type of sports activity due to COVID-19: hypochondriasis and the intention of continuous participation in sports. *Int. J. Environ. Res. Public Health* 17:4871. 10.3390/ijerph17134871 32640684 PMC7369974

[B13] Chtourou H. Trabelsi K. H’mida C. Boukhris O. Glenn J. Brach M. (2020). Staying physically active during the quarantine and self-isolation period for controlling and mitigating the COVID-19 pandemic: a systematic overview of the literature. *Front. Psychol.* 11:1708. 10.3389/fpsyg.2020.01708 33013497 PMC7466737

[B14] Clemente-Suárez V. J. Fuentes-García J. P. de la Vega R. Martínez-Patiño M. J. (2020). Modulators of the personal and professional threat perception of Olympic athletes in the actual COVID-19 crisis. *Front. Psychol.* 11:1985. 10.3389/fpsyg.2020.01985 32849157 PMC7419607

[B15] Constant A. Conserve D. Gallopel-Morvan K. Raude J. (2020). Socio-cognitive factors associated with lifestyle changes in response to the COVID-19 epidemic in the general population: results from a cross-sectional study in France. *Front. Psychol.* 11:2407. 10.3389/fpsyg.2020.579460 PMC7550454 33132989

[B16] Crespo M. Cruzado J. A. (1997). La evaluación del afrontamiento: adaptación española del cuestionario COPE con una muestra de estudiantes universitarios. *Análisis y Modificación de Conducta* 23 797–830.

[B17] Díaz D. Rodríguez R. Blanco A. Moreno B. Gallardo I. Dierendonck D. (2006). Adaptación española de las escalas de bienestar psicológico de Ryff. *Psicothema* 18 572–577. 17296089

[B18] Ferreira-Júnior J. Freitas E. Chaves S. (2020). Exercise: a protective measure or an “open window” for COVID-19? a mini review. *Front. Sport Active Living* 2:61. 10.3389/fspor.2020.00061 PMC7739719 33345052

[B19] Filho A. Miranda T. de Paula C. Barsanulfo S. Teixeira D. Monteiro D. (2020). COVID-19 and quarantine: expanding understanding of how to stay physically active at home. *Front. Psychol.* 11:566032. 10.3389/fpsyg.2020.566032 33192841 PMC7658189

[B20] Fu W. Wang C. Zou L. Guo Y. Lu Z. Yan S. (2020). Psychological health, sleep quality, and coping styles to stress facing the COVID-19 in Wuhan, China. *Trans. Psychiatry* 10:225. 10.1038/s41398-020-00913-3 32647160 PMC7347261

[B21] García-Naveira A. Locatelli L. (2014). “Psychological benefits in physical activity and sports,” in *Psychological health and needs research developments*, ed. Wolfe R. (Hauppauge, NY: Nova).

[B22] Ingram J. Maciejewski G. Hand C. (2020). Changes in diet, sleep, and physical activity are associated with differences in negative mood during COVID-19 lockdown. *Front. Psychol.* 11:2328. 10.3389/fpsyg.2020.588604 PMC7492645 32982903

[B23] Jaenes J. C. García-González P. González-López J. Costa-Agudo M. García-Ordoñez J. Mehrsafar A. (2020). ¿ Es el entrenamiento, un moderador de reacciones emocionales en el confinamiento por COVID-19, en deportistas de alto rendimiento? *Revista Andaluza de Medicina del Deporte* 13 120–121. 10.33155/j.ramd.2020.06.003

[B24] Jimeìnez-Pavoìn D. Carbonell-Baeza A. Lavie C. J. (2020). Physical exercise as therapy to fight against the mental and physical consequences of COVID-19 quarantine: special focus in older people. *Prog. Cardiovas. Dis.* 63 386–388. 10.1016/j.pcad.2020.03.009 PMC7118448 32220590

[B25] Jukic I. Calleja-Gonzaìlez J. Cos F. Cuzzolin F. Olmo J. Terrados N. (2020). Strategies and solutions for team sports athletes in isolation due to COVID-19. *Sports* 8:56. 10.3390/sports8040056 PMC7240607 32344657

[B26] Jungmann S. Witthöft M. (2020). Health anxiety, cyberchondria, and coping in the current COVID-19 pandemic: which factors are related to coronavirus anxiety? *J. Anxiety Disord.* 73:102239. 10.1016/j.janxdis.2020.102239 32502806 PMC7239023

[B27] Lesser I. Nienhuis C. (2020). The impact of COVID-19 on physical activity behavior and well-being of Canadians. *Int. J. Environ. Res. Public Health* 17:3899. 10.3390/ijerph17113899 32486380 PMC7312579

[B28] Liu S. Lithopoulos A. Zhang C. Garcia-Barrera M. Rhodes R. (2021). Personality and perceived stress during COVID-19 pandemic: testing the mediating role of perceived threat and efficacy. *Person. Indiv. Differ.* 168:110351. 10.1016/j.paid.2020.110351 PMC7442020 32863508

[B29] López-Bueno R. Calatayud J. Andersen L. L. Balsalobre-Fernández C. Casaña J. Casajús J. A. (2020a). Immediate Impact of the COVID-19 confinement on physical activity levels in spanish adults. *Sustainability* 12:5708. 10.3390/su12145708

[B30] López-Bueno R. Calatayud J. Casaña J. Casajús J. A. Smith L. Tully M. A. (2020b). COVID-19 confinement and health risk behaviors in Spain. *Front. Psychol.* 11:1426. 10.3389/fpsyg.2020.01426 32581985 PMC7287152

[B31] López-Bueno R. Calatayud J. Ezzatvar Y. Casajús J. A. Smith L. Andersen L. L. (2020c). Association between current physical activity and current perceived anxiety and mood in the initial phase of COVID-19 confinement. *Front. Psychiatry* 11:729. 10.3389/fpsyt.2020.00729 PMC7390883 32793013

[B32] López-Sánchez G. F. López-Bueno R. Gil-Salmerón A. Zauder R. Skalska M. Jastrzȩbska J. (2020). Comparison of physical activity levels in spanish adults with chronic conditions before and during COVID-19 quarantine. *Eur. J. Public Health* 2020:ckaa159. 10.1093/eurpub/ckaa159 32761181 PMC7454536

[B33] Maugeri G. Castrogiovanni P. Battaglia G. Pippi R. D’Agata V. Palma A. (2020). The impact of physical activity on psychological health during COVID-19 pandemic in Italy. *Heliyon* 6:04315. 10.1016/j.heliyon.2020.e04315 32613133 PMC7311901

[B34] Mon-López D. García-Aliaga A. Ginés A. Muriarte D. (2020). How has COVID-19 modified training and mood in professional and nonprofessional football players? *Physiol. Behav.* 227:113148. 10.1016/j.physbeh.2020.113148 PMC7445487 32858031

[B35] Moscoso-Sánchez D. (2020). El contexto del deporte en España durante la crisis sanitaria de la COVID-19. *Sociol. del Deporte* 1 15–19. 10.46661/socioldeporte.5000

[B36] Pfefferbaum B. North C. S. (2020). Mental health and the COVID-19 pandemic. *N. Eng.J. Med.* 383 510–512. 10.1056/NEJMp2008017 32283003

[B37] Pieh C. Budimir S. Probst T. (2020). The effect of age, gender, income, work, and physical activity on mental health during coronavirus disease (COVID-19) lockdown in Austria. *J. Psych. Res.* 136 1–9. 10.1016/j.jpsychores.2020.110186 32682159 PMC7832650

[B38] Rettie H. Daniels J. (2020). Coping and tolerance of uncertainty: predictors and mediators of mental health during the COVID-19 pandemic. *Am.Psychol.* 2020:32744841. 10.1037/amp0000710 32744841

[B39] Rossi R. Socci V. Talevi D. Mensi S. Niolu C. F. (2020). COVID-19 pandemic and lockdown measures impact on mental health among the general population in Italy. *Front. Psych.* 11:790. 10.3389/fpsyt.2020.00790 32848952 PMC7426501

[B40] Schinke R. Papaioannou A. Henriksen K. Si G. Zhang L. Haberl P. (2020). Sport psychology services to high performance athletes during COVID-19. *Int. J. Sport Exercise Psychol.* 18 269–272. 10.1080/1612197X.2020.1754616

[B41] Schnitzer M. Schöttl S. Kopp M. Barth M. (2020). COVID-19 stay-at-home order in tyrol, austria: sports and exercise behavior in change? *Public Health* 185 218–220. 10.1016/j.puhe.2020.06.042 32659514 PMC7318923

[B42] Sherwin I. (2017). Commentary: from mental health to mental wealth in athletes: looking back and moving forward. *Front. Psychol.* 8:693. 10.3389/fpsyg.2017.00693 28539896 PMC5423944

[B43] Slimani M. Paravlic A. Mbarek F. Bragazzi N. Tod D. (2020). The relationship between physical activity and quality of life during the confinement induced by COVID-19 outbreak: a pilot study in Tunisia. *Front. Psychol.* 11:1882. 10.3389/fpsyg.2020.01882 32849104 PMC7427614

[B44] Van Dierendonck D. (2004). The construct validity of Ryff’s Scale of psychological well-being and its extension with spiritual well-being. *Person. Indiv. Differ.* 36 629–644. 10.1016/s0191-8869(03)00122-3

[B45] Woods J. Hutchinson N. Powers S. Roberts W. Gomez-Cabrera M. Radak Z. (2020). The COVID-19 pandemic and physical activity. *Sport Med. Health Sci.* 2 55–64. 10.1016/j.smhs.2020.05.006 PMC7261095 34189484

[B46] Xiang M. Zhang Z. Kuwahara K. (2020). Impact of COVID-19 pandemic on children and adolescents’ lifestyle behavior larger than expected. *Prog. Cardiovas. Dis.* 63 531–532. 10.1016/j.pcad.2020.04.013 32360513 PMC7190470

[B47] Xionga J. Lipsitzc O. Nasric F. Luic L. Gillc H. Phanc (2020). Impact of COVID-19 pandemic on mental health in the general population: a systematic review. *J. Affec. Disord.* 277 55–64. 10.1016/j.jad.2020.08.001 32799105 PMC7413844

[B48] Ye Z. Yang X. Zeng C. Li X. Wang Y. Shen Z. (2020). Resilience, social support, and coping as mediators between COVID-19-related stressful experiences and acute stress disorder among college students in China. *Appl. Psychol. Health Well-Being* 10:1111. 10.1111/aphw.12211 32666713 PMC7405224

